# A Review on the Efficacy of Extraosseous Local Infiltration of Multimodal Drug Cocktail for Pain Management After Total Knee or Hip Arthroplasty

**DOI:** 10.7759/cureus.30451

**Published:** 2022-10-19

**Authors:** Ankur Salwan, Gajanan L Pisulkar, Shounak Taywade, Abhiram A Awasthi, Amit Saoji, Vivek H Jadawala, Parth Shah, Sanjay V Deshpande

**Affiliations:** 1 Orthopaedics and Traumatology, Datta Meghe Institute of Medical Sciences, Wardha, IND; 2 Orthopedics and Traumatology, Datta Meghe Institute of Medical Sciences, Wardha, IND

**Keywords:** rehabilitation, epidural injections, post op pain management, total joint arthroplasty, intra-articular cocktail infiltration

## Abstract

Total knee arthroplasty (TKA) patients express minimal comfort regarding postoperative pain management. The use of parenteral opioids or epidural analgesia may have unfavorable adverse impacts that interfere with quick healing and rehabilitation. It is uncertain if periarticular multimodal drug injections (PMDI) are effective at easing pain following total knee or total hip arthroplasty (THA). We conducted this study to assess the effectiveness of PMDI following TKA or THA. Articles were sourced using the following keywords on Pubmed, Google scholar, and the Web of Science: multimodal drug cocktail in total knee arthroplasty OR hip arthroplasty, periarticular injections AND multimodal drug cocktail, epidural versus periarticular injections AND pain management after total joint arthroplasty. After screening 438 articles and abstracts, 200 pertinent studies were found, of which a total of 10 articles were included in the study. From this review, we want to conclude that despite the various ways to address postoperative pain, there is no acknowledged gold standard for postoperative pain management following total joint arthroplasty. To reduce narcotic intake and prevent narcotic-related adverse reactions, multimodal techniques utilizing regional anesthetics appear to be on the rise such as periarticular injections, or patient-controlled analgesia with or without femoral nerve block. Even though the ideal duration and kind of medications are unclear, preoperative pain management or preemptive analgesia with anti-inflammatory drugs and opioid analgesics seem to be useful in lowering postoperative pain.

## Introduction and background

For patients with chronic degenerative joint disorders of the hip or knee, total joint arthroplasties (TJA) such as total knee arthroplasty (TKA) or total hip arthroplasty (THA) are considered the most effective treatment option [1]. Following a hip and knee arthroplasty, patients may experience severe postoperative pain that often necessitates hospitalization for five to 10 days to receive appropriate analgesia. Long-term hospital stays and immobility in bed may be conducive to nosocomial infections and deep vein thrombosis (DVT) [2]. Physical activity triggers pain, which then limits functional ability [3]. Pain and function are interconnected. After THA, pain management strategies are crucial for an early improvement in postoperative quality of life and prompt postoperative recovery [4]. Anesthetic infiltration at the operative area is part of pain control therapy [5]. To reduce perioperative morbidity and hasten recovery after total joint arthroplasty, anesthetic procedures, perioperative pain control, and functional management are frequently disregarded [6]. In comparison to systemic methods, recent studies have demonstrated that intraarticular infiltration and injection or infusion of local anesthesia in the surgical area are more successful in reducing postoperative pain and narcotic intake [7]. Parenteral opioids or epidural analgesia use may have unfavorable adverse reactions interfering with initial recovery and rehab. The local infiltration of an analgesic mixture can prevent these negative effects [8]. Subsequent studies in this field will render painful, challenging recoveries following total knee and hip arthroplasty a distant memory [9].

Over the past 10 years, the pain management strategy following THA and TKA has undergone numerous changes [10]. Following TKA, there are several choices for postoperative pain management, but each of them has drawbacks [11]. Cardiorespiratory problems may result from postoperative discomfort that is not adequately managed [12]. Postoperative discomfort with associated immobilization and splinting of joints [13]. Through a variety of processes and by focusing on multiple facets of the pain perception pathway, multimodal pain management is crucial for enhancing outcomes and optimizing patient outcomes about total joint arthroplasty [14]. The cocktail's ingredients are always local anesthetics (ropivacaine and bupivacaine), epinephrine, steroids, opioids, and nonsteroidal anti-inflammatory medications, even if the gold standard for the cocktail formula has not yet been established. For rapid rehabilitation and improved functional results, postoperative pain must be effectively managed [15]. The first nine to 12 hours after surgery under general anesthesia are when patients feel the most discomfort. It is general knowledge that if the pain is managed at this time by a long-acting analgesic, managing pain afterward is simpler, in part because worry has been reduced. Recent studies have evaluated the effectiveness of local anesthetics in relieving pain during wound infiltration [16]. Systemic analgesics, ongoing peripheral nerve blocks, and most recently high-volume local infiltration analgesia have all helped to enhance post-operative pain control in TKA during the last several years, resulting in shorter hospital stays and perioperative complications. In the early postoperative phase of TKA, high-volume local infiltration analgesia with a single-shot administration of local anesthetics directly to the wound along with postoperative injections to the wound is effective but is constrained by the local anesthetic's duration of action [17].

It is also unclear which area of the wound would aid the infiltration of a local anesthetic to effectively lessen post-operative pain because the high-volume infiltration technique contains infiltration of all tissues incised or instrumented in the procedure (i.e., joint capsule, soft tissue, as well as subcutaneous tissue). In the early postoperative period, high-volume local infiltration analgesia could be able to give enough post-operative analgesia to ease mobilization and enable early rehabilitation with no need for rescue opioid analgesics.

## Review

**Figure 1:**
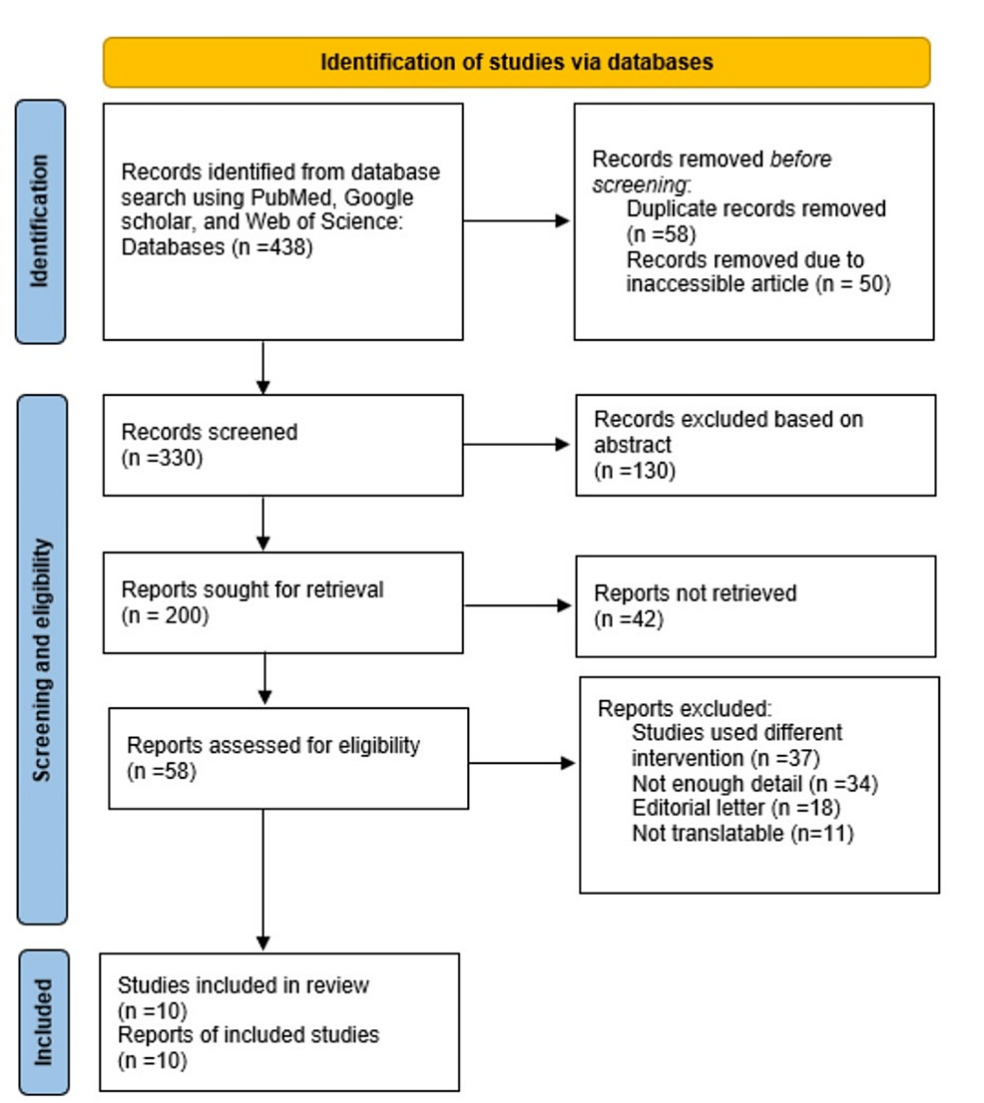
PRISMA flow diagram outlining the review strategy PRISMA: Preferred Reporting Items for Systematic Reviews and Meta-Analyses

We looked through the databases of PubMed, Google Scholar, and the Web of Science. The following phrases were used by an independent reviewer to search the references lists of the original and review publications: multimodal drug cocktail in total knee arthroplasty OR hip arthroplasty, periarticular injections AND multimodal drug cocktail, epidural versus periarticular injections AND pain management after total joint arthroplasty. While looking for articles, no year limits were used. After screening 438 articles and abstracts, 200 pertinent items were found. Of these, 10 publications were eventually included after 200 eligible articles underwent full-text analysis. We evaluated each article's caliber and key research facets. The factors included specified post-operative pain management and rehabilitation criteria. We collected and evaluated data from the 10 carefully chosen articles. Figure 1 presents an outline of the review strategy.

Epidural analgesia

It is normal practice to employ epidural anesthesia in addition to general anesthesia. Although it is becoming less prevalent as anesthesiologists refine adductor canal blocks, it is frequently used when both knees are replaced at the same time [18]. An advantage of epidural anesthesia is that it leaves a catheter in place, making it simple to top it off and allowing use for up to three days following surgery. The drawbacks are caused by the difficulty of insertion and the inconsistent application. When used properly, epidural anesthesia provides great pain relief. On the other hand, epidural anesthesia has a reputation for being unreliable. It typically affects one limb more than the other, making it difficult to spread to the necessary side. You might need to roll to the side for a while to let the anesthesia spread across the area to attempt and stop the spread and receive the anesthetic where it is needed. Epidural analgesia with local anesthetics is known to cause lower limb motor paralysis. It causes delays in the postoperative rehabilitation process and may call for the temporary or permanent cessation of the epidural infusion, which is painful, uncomfortable for the patient, and unsatisfactory [19]. Hence, local blocks are being encouraged.

Periarticular injection

Periarticular injections with long-acting liposomal bupivacaine are an effective technique to lessen preoperative discomfort following total hip/knee arthroplasty in the lower limb, while also lowering opiate use and its negative effects. It has the potential to lessen the negative effects of opiates and peripheral nerve blocking while enhancing muscle control during recovery [20]. The infiltration technique must be carefully watched to prevent leaking from the soft tissues, and intravascular injection should be avoided. It is necessary to concentrate the periarticular injection on the knee and hip's most innervated regions to benefit from it most. This can aid in the management of postoperative pain following total joint arthroplasty.

Efficacy of the multimodal drug cocktail

Total hip replacement has developed over the past 40 years into a highly effective treatment for enhancing the quality of life for patients with end-stage degenerative joint disease [21]. The most frequent arthroplasty treatment performed worldwide is TKA, and its use is rising quickly [22]. Joint replacement has been demonstrated to lessen discomfort, increase functioning, and improve quality of life [23]. The majority of earlier research has concentrated on the risk factors for radiographic osteoarthritis (OA), despite the fact that symptomatic knee OA is frequent, causes significant impairment, and utilizes a lot of medical resources. Not every risk factor for radiographic OA is a reliable indicator of joint pain [24]. Following TKA, postoperative discomfort is a significant problem. In 60% of patients, it is extreme, and in 30%, it is medium. It amplifies reflex reactions when untreated, which can result in significant consequences such as thromboembolism, hyperdynamic circulation, and increased oxygen consumption [25]. Due to the variables at play (temperature, exposure time, and nerve diameter), the block's duration is uncertain, however, it is typically measured in weeks or months. The use of cryoanalgesia in TKA is supported by scant clinical data [26]. Scientific proof and treatment regimen of postoperative pain treatment aims to give the best analgesia with the fewest side effects. Although there aren't yet any studies specific to THR, there are a variety of analgesic treatments that might be effective. These include ketamine, gabapentin, and local anesthetic injection into surgical wounds. Until more information is available, these medications cannot be advised for analgesia following THR [27]. Quantifying the anticipated number of revisions to arthroplasty procedures for the knee and hip in the future is difficult [28]. Furthermore, in comparison to primary TKA, upwards of twice as many patients undergo revision of TKA surgery and experience persistent discomfort. Another reason local infiltration analgesia (LIA) is more efficient could be due to the medicines' improved nerve block action. Non-steroidal anti-inflammatory drugs (NSAIDs) have a more effective analgesic effect when administered intraarticularly as opposed to systemically via intravenous (IV) injection [29].

Applying a pressure bandage and an ice pack to the wound area may aid to extend the LIA's duration of effect and so enhance the postoperative function of TKA patients [30]. Gomez-Cardero et al. concluded in their study that after TKA, using the continuous infusion pump to manage post-operative pain reduces the amount of pain experienced, and the need for opioid painkillers at the time of peak pain, as well as the average time spent in the hospital [31]. Affas et al. in their review said that the effectiveness of pain reduction following LIA for knee arthroplasty is comparable to what follows a femoral block, and the plasma concentration of ropivacaine following LIA is somewhat higher than that following a femoral block [32]. Vaishya et al. in their clinical trial concluded that following TKA, the LIA approach combined with multimodal medications can dramatically enhance pain control both at rest and when moving, leading to higher patient contentment. Additionally, it lessens the need for analgesics like morphine [33]. Min et al. concluded in their review that multimodal pain control has grown to be a crucial component of postoperative therapy for those getting TJA. The idea behind multimodal therapy is to employ many methods that target various pain pathways, resulting in more effective pain management with fewer consequences [34]. Zhang et al., in their randomized trial, concluded that depending on their results more morphine given to a multimodal cocktail did not substantially lower the post-operative pain levels, but it did lower the systemic opioids intake in TKA [35].

Chen et al. in their retrospective study observed that there were numerous postoperative usages of modes of analgesia for patients who have undergone TKA to control their pain. The type of operation, the level of surgery, the length of the surgery, and the anesthesia technique may all need to be taken into account when deciding whether to utilize analgesics [36]. Martin et al. in their study stated that the post-operative healing following in total knee replacement has been revolutionized by the use of a combination of an efficient, technologically well-driven periarticular cocktail injection, a multimodal supplemental pain program, as well as the application of tranexamic acid to limit bleeding. When it comes to pain control, the multimodal approach has exceptionally high patient contentment [37]. Additionally, patients may be able to prevent the necessity and dangers of a subsequent peripheral nerve block surgery. In particular, if the facility lacks a dedicated regional anesthetic block team, eliminating the necessity for a preoperative procedure may decrease the amount of time an operating room is in use (Table 1 ) [38].

**Table 1:** Review of the available studies RCT:  Randomized control trial, VAS: Visual analog scale, EPA: Epidural anesthesia, LIA: Local infiltration anesthesia, TKA: Total knee arthroplasty, TKR: Total knee replacement, PNB: Peripheral nerve block, LBPAI: Liposomal bupivacaine peri-Articular injection, ACB:  Adductor canal block

Sr. No	Author	Year	Study Type	Study Sample	Intervention	Result	Analysis
1	Snyder et al. [5]	2016	RCT	70	Bupivacaine liposomal suspension injection & concentrated cocktail mixture injection	Patients who were given bupivacaine liposomal suspension expressed greater pain relief satisfaction	Injection of bupivacaine liposomal suspension is superior to injection of a concentrated cocktail
2	Andersen et al. [7]	2007	RCT	80	Intraarticular technique vs Epidural infusion	The use of narcotics was decreased. Although pain levels were similar in both groups, one group's pain dramatically decreased when therapy ended, and their length of stay in the hospital also decreased	The intraarticular method performed notably better than the epidural infusion
3	Nakai T et al. [4]	2013	RCT	54	Local periarticular injection versus no injection	On the day of the procedure, VAS in the injection group was noticeably low. There was no evidence of harm to the cardiac or nervous system.	Following THA, intraoperative periarticular injection with multimodal medications can greatly lessen the pain on the day of surgery with no obvious complications.
4	Ogonda et al. [21]	2005	RCT	219	Minimally invasive total hip arthroplasty	There was no discernible difference in postoperative hematocrit, the need for blood transfusions, pain scores, or painkiller usage	Although it is a safe and reliable treatment, there is no discernible advantage over a typical 16 cm incision
5	Alsheikh KA et al. [18]	2020	Observational Study	80	Continuous epidural analgesia vs Adductor canal block	Patients with ACB had better pain relief, and post-operative degree of movement with less postoperative blood drain output	After TKR, ACB as postoperative analgesia gives superior results in terms of promoting early functional recovery and mobility, and helps avoid significant postoperative sequelae.
6	Vaishya et al. [33]	2015	RCT	80	Local infiltration analgesia vs Saline injections	Patients with LIA had better results	Following TKA, the LIA approach with multimodal medications can dramatically improve pain control both at rest and when moving, leading to improved patient satisfaction.
7	Martin et al. [37]	2020	RCT	64	Periarticular intra-operative injection containing ropivacaine, fentanyl, clonidine, cefuroxime, and epinephrine (Group A) vs Plain ropivacaine (Group B)	The periarticular mixture improved the functional recovery and pain scores of the patients	Following TKR, periarticular cocktail injection considerably reduces the need for postoperative analgesia while simultaneously increasing patient satisfaction, with no obvious hazards.
8	Tian et al. [29]	2020	Observational study	206	Peripheral nerve block (PNB) vs Local infiltration analgesia (LIA)	There were no discernible differences between the two groups in terms of pain score	When compared to LIA, which consumed fewer opioids without significantly differing, LIA offered superior postoperative analgesia.
9	Andersen LØ et al. [17]	2019	RCT	16	Patients undergoing bilateral knee arthroplasty with high-volume local infiltration analgesia with ropivacaine in one knee and saline infiltrated into the other knee	The knees infiltrated with local analgesia had considerably lower VAS pain levels than the knees infiltrated with saline. A decrease in VAS pain scores was seen 24 hours after surgery in both groups, with no statistically significant difference between the groups receiving local anesthetic injections of ropivacaine or saline.	The local analgesia group only showed pain relief in the first 24 hours; further administration of bolus drug didn’t yield any beneficial effect.
10	Than et al. [38]	2021	RCT	56	Liposomal bupivacaine peri-articular injection (LB-PAI) vs Single-shot adductor canal block (ACB) using bupivacaine	On postoperative days 0, 1, and 2, the LB-PAI group showed a trend toward reduced average pain scores when compared to the ACB group	Between LB-PAI and a single-shot ACB, similar postoperative pain management, functional results, and opioid use were observed

## Conclusions

Our findings suggest that there are a variety of perioperative analgesic utilization modes for pain management in patients. The decision about analgesic use might need to take into consideration the surgery type, surgery level, surgery duration, and anesthesia method. The findings of our study unequivocally demonstrate that periarticular cocktail injection in TKR helps in both early healing and rehabilitation in addition to pain relief. Future studies should take measures to prevent nausea and vomiting. The use of regional anesthesia and multimodal pain management strategies that forgo the needless use of drugs can help achieve painless THA or TKA. In the coming years, local, periarticular injections will play a significant role in these programs. Longer-acting injectable medicines still need to be discovered through additional studies. Further clinical evidence is needed to support the development of guidelines for drug use. In the future, larger studies are warranted to help expand our understanding in this regard.
